# A Qualitative Exploration of the Role of Vape Shop Environments in Supporting Smoking Abstinence

**DOI:** 10.3390/ijerph15020297

**Published:** 2018-02-09

**Authors:** Emma Ward, Sharon Cox, Lynne Dawkins, Sarah Jakes, Richard Holland, Caitlin Notley

**Affiliations:** 1 Norwich Medical School, University of East Anglia, Norwich Research Park, Norwich NR4 7TJ, UK; c.notley@uea.ac.uk; 2 Centre for Addictive Behaviours Research, School of Applied Sciences, London South Bank University, 103 Borough Road, London SE1 0AA, UK; coxs15@lsbu.ac.uk (S.C.); dawkinl3@lsbu.ac.uk (L.D.); 3 New Nicotine Alliance, 8 Northumberland Avenue, London WC2N 5BY, UK; sarah@nnalliance.org; 4 George Davies Centre, Leicester Medical School, University of Leicester, Lancaster Road, Leicester LE1 7RH, UK; rch23@leicester.ac.uk

**Keywords:** electronic cigarettes, vape shops, smoking cessation, relapse prevention, stop smoking services

## Abstract

E-cigarettes are the most popular method of quitting smoking in England and most are purchased in specialist vape shops. This qualitative study explores how the vape shop environment is experienced by quitters to support smoking abstinence. Semi-structured qualitative interviews were conducted to elicit experiences of e-cigarette use, including experiences of vape shops, in 40 people who had used e-cigarettes in a quit attempt. Observations of six shops in a range of locations were also undertaken. Interview and observation data were analysed using inductive thematic analysis and triangulated. At an individual level, smoking abstinence was supported through shop assistants’ attempts to understand customers’ smoking preferences in order to: (i) tailor advice about the most appropriate product; and (ii) offer an ongoing point of contact for practical help. At an interpersonal level, shops offered opportunity to socialise and reinforce a vaping identity, although the environment was perceived as intimidating for some (e.g., new and female users). At a structural level, shops ensured easy access to products perceived to be good value by customers and had adapted to legislative changes. Vape shops can provide effective behavioural support to quitters to maintain smoking abstinence. Health professionals could capitalise on this through partnership working with shops, to ensure best outcomes for clients wanting to use e-cigarettes to quit smoking.

## 1. Introduction

For smokers, quitting combustible tobacco is the best way to improve health outcomes [1], but an estimated 90% of traditional quit attempts end in relapse [2]. E-cigarettes are now the most popular method of quitting smoking in England [3] and they can be as effective, if not more effective, than other methods [4,5,6]. Smokers are addicted to the nicotine in tobacco, but smoking is also a complex psychosocial behaviour incorporating habits, beliefs, and identity [7]. Nicotine replacement therapy (NRT) may fail to address these behavioural and social aspects of smoking, whereas e-cigarettes relieve nicotine cravings and replace the behavioural and social aspects [8,9,10]. Vapers also report that e-cigarettes have their own unique pleasurable qualities [8,10,11,12] and advantages over tobacco such as flavours, less stigma, and lower cost [8,9]. Research reviews indicate that e-cigarettes are considerably safer than smoking [13,14], and their potential usefulness as a harm reduction measure has been acknowledged in UK policy [15] and practice guidance [16,17].

Many Stop Smoking Services (SSS) identify themselves as ‘e-cigarette friendly’ [18,19], but advice about specific vaping products is limited and there are no e-cigarettes currently licensed for medicinal use. National guidance recommends that SSS familiarise themselves with e-cigarettes through visiting a ‘reputable retailer’ (p. 10) [16]. How many SSS actively do this is unknown, and SSS advisors vary in their response to the commercial success of e-cigarettes based on their personal beliefs about nicotine addiction and normalisation, and the tobacco industry [20]. This highlights a need for research into the possible role of vape shops in supporting quitters so that health professionals can make informed decisions when referring clients to shops.

Vape shops are often the ‘frontline’ for cessation support [21] because most people who choose e-cigarettes to quit smoking do so without the support of SSS. Specialist vape shops are by far the most popular places that the estimated 2.9 million vapers in Britain [22] purchase e-cigarettes [3]. Currently, there are approximately 2000 shops in the UK and the industry is estimated to be worth over £600 million annually in the UK alone [23]. As yet, vape shops do not require a licence and instead the industry has its own trade associations [24,25] and code of conduct [26]. UK shops are obliged to comply with the Tobacco and Related Products Regulations 2016 (TRPR), an EU directive, which sets out restrictions on e-liquid and tank sizes, nicotine strengths and advertising, and new requirements for notification of ingredients, emissions and product labelling. The legislation has been met with concern from vapers [27], industry [28], and the scientific community [29], with suggestion that it may negatively affect numbers of people switching from tobacco to vaping by increasing vaping costs and reducing accessibility, choice and effectiveness. The current study adds insight into how vape shops have reacted to the legislation. 

There is some evidence that vape shops can influence smoking cessation success rates. In a prospective real world study of seventy-one tobacco quitters who bought their first e-cigarette from a vape shop, 41% were still abstinent from tobacco after 12 months [30]. The authors suggest that a ‘combination of high quality e-vapour products together with personalized e-cig support and advice at vape shops promotes high success rates’ and that ‘vape shops may become valuable allies in the fight against smoking’ [30] (pp. 3435–3436). It is important to acknowledge that this study is limited by having no control group, having a small self-selecting sample, and using self-report measures. However, it does hint that interaction with a vape shop could promote success rates potentially higher than those achieved with traditional SSS methods [31], warranting further exploration of their role in helping people quit smoking.

Whilst there seems to be evidence that some consumers would appreciate traditional smoking cessation advice from vape shops [9], most vapers appear to value shop advice relating to vaping products [9,32]. Vapers report that an unsatisfying vaping setup, device malfunction or a lack of access to vaping consumables, can trigger smoking relapse [33]. Therefore, product support offered by vape shops in these areas may help sustain smoking abstinence.

Existing US vape shop research shows that most staff have personal experience of using e-cigarettes to quit tobacco, hold strong beliefs regarding the health benefits and safety of e-cigarettes, and their ability to help smokers quit [34,35,36]. Vape shop observations showed that they extensively promoted their products and offered cessation related product advice [36,37], as well as offering a place of recreation [38]. One study showed that the most successful shops were more likely to be endorsed in online reviews for: helping to fix devices, having ‘helpful/patient/respectful staff’, and a ‘bar type’ environment [39] (p. 1). The existing research indicates that vape shops do not passively sell e-cigarettes like other retailers [40], but instead may offer ‘expert by experience’ product advice in an enticing environment. It is not clear, however, how the shop environment is experienced by tobacco quitters to support them to remain smoke free. This study aims to address this research gap by triangulating qualitative data collected through vape shop observations and quitters’ experiences of vape shops.

## 2. Materials and Methods

The data drawn upon to answer the research question ‘what role does the vape shop environment have in supporting smoking abstinence?’ are taken from a wider qualitative study (ECtra Study) exploring patterns of e-cigarette use in relation to preventing smoking relapse [41]. Two data sources were used in analysis: semi-structured interviews and observations. The study received ethical approval from the UEA Faculty of Medicine and Health Sciences Research Ethics Committee (project reference: 2015/2016-144).

### 2.1. Interview Sample, Recruitment, and Data Collection

In-depth semi-structured interviews were conducted between September 2016 and May 2017 with forty people (20 male and 20 female) who had used e-cigarettes to try to quit smoking. Participants were recruited through word of mouth, local press articles, university bulletins, and social media, and were purposively sampled for maximum variation in demographic characteristics.

Participants gave written consent before participating in a face-to-face (35) or telephone/Skype interview (5). Interviews were recorded, transcribed verbatim, and anonymised. The anonymous participant code used to reference quotes refers to the participant’s gender and age (e.g., ‘F24’ is shorthand for ‘female aged 24 years’). In line with the objectives of the wider ECtra Study, the topic guide covered all aspects of e-cigarette use, including experiences of vape shops. Participants were free to discuss any vape shop they had experienced, and although it is possible that they had visited the observation sample shops due to sampling from the same geographical areas, this was not recorded or used in analysis. Interview participants are referred to as ‘vapers’ in the results section.

### 2.2. Observation Sample, Recruitment and Data Collection

It became apparent from the interview data that vape shops had an important role in the success of many participants’ smoking quit attempts. The qualitative approach used in the ECtra Study allowed flexibility and iterative use of appropriate methods to explore avenues of inquiry further, such as the supportive role of vape shops, prompting an amendment to the protocol to include observations. Observations were conducted in six vape shops recruited by researchers through informal discussions with shop managers and staff, purposely sampled to include a range of urban city (4) and rural town (2) locations. All shop staff who were approached across these six shops, agreed to participate (9). Observations took place from May to September 2017 at a variety of times during the week and weekends. The shops were based in London (3) and East Anglia (3).

With full written consent from the manager and participating staff, a researcher sat in situ for around 3 h per visit taking handwritten notes about the shop context, the interactions between customers and staff, and conversations with participating shop staff. Formal written consent was not required from customers as minimal data were collected about them: observations focused on the support shop staff gave to customers, rather than the characteristics of the customer or what the customer did. Instead, a notice was displayed in the shop informing customers that an observation was taking place, staff checked with customers that they agreed to being observed for research purposes, and an information sheet aimed at customers was made available. Immediately following each observation, notes were typed up by the researcher who added their own thoughts and reflections. Notes were anonymised and each shop was randomly assigned a code.

### 2.3. Analysis

**Figure 1:**
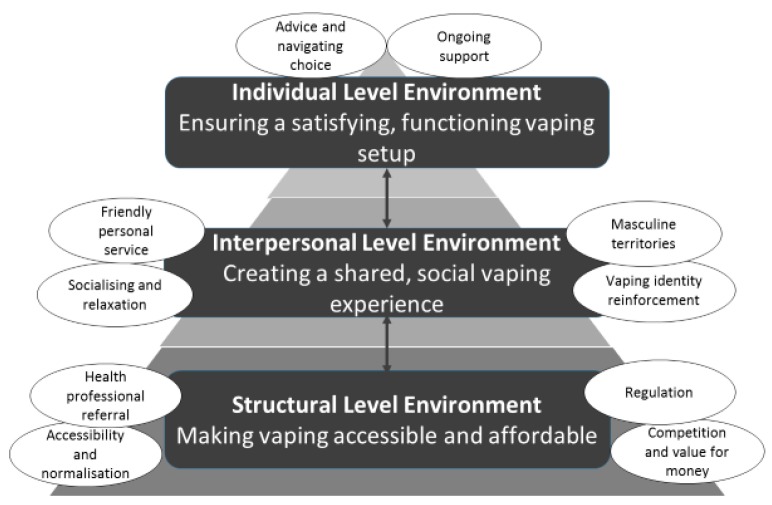
Thematic structure relating to the role the vape shop environment has in supporting smoking abstinence.

Interview transcripts and observation notes were uploaded onto NVivo11 qualitative software. Interview transcripts were broad coded for data relating to vape shops by coding the part of the transcript relating to the questions about shops. In addition, a word search was performed to capture data relating to shops in other parts of the interview. The interview data extracts relating to shops and observation notes were coded using an established inductive thematic analysis methodology [42]. E.W. coded interview and observation data for latent and semantic content. Codes were reviewed and sorted into subthemes and overarching themes by E.W. in discussion with C.N. The themes were written up and triangulated with illustrative quotes from both interviews and observations for each identified theme. This analytical write up was critically reviewed by C.N. and S.C. resulting in a comprehensive interpretation of the data in relation to the research question. The final thematic structure agreed by E.W., C.N., and S.C. is shown in Figure 1.

## 3. Results

Vapers’ ages ranged from 21 to 70 years (mean 41, SD 13.97). All vapers identified as White British or European, 16 were employed in managerial, professional or technical occupations [43], and all were recruited in England, 11 from rural areas, with 33 resident in the East Anglia region. Vaping experience varied from 2 weeks to 7 years. Thirty-one participants were vaping and abstinent from tobacco (19 reported lapses), six participants had relapsed (5 dual using both tobacco and vaping), and three were no longer using either e-cigarettes or tobacco. Demographics were not collected for observation shop staff. Overarching themes relating to structural, interpersonal, and individual environments were identified (Figure 1) and are discussed in turn using illustrative quotes from both observation and interview data. Some quotes have been edited to improve readability by removing repeated/redundant words and discourse markers (e.g., ‘um’, ‘er’).

### 3.1. Structural Level Environment—Making Vaping Accessible and Affordable

#### 3.1.1. Accessibility and Normalization

Many vapers commented on the increased accessibility of vape shops in commercial environments such as highstreets. The presence of vape shops in these everyday environments normalised vaping for some quitters, making it appear socially acceptable and low risk to health, prompting them to try e-cigarettes:

*I think you just see a lot of people using [e-cigarettes] now and there’s a lot of shops selling them. Certainly, in this area where I am, I only live in a small town, and there’s two shops that sell vaping supplies, so it’s really common. I went into a shop and you could actually try them there. So I tried them in the shop and then I bought one.*

(F40)

#### 3.1.2. Competition and Value for Money

Nearly every vaper interviewed commented on the reduced cost of vaping compared to smoking as a key benefit. Shops reinforced this message via posters outlining the cost of vaping vs. smoking. One shop assistant described how he explained to new switchers: *‘how much [they] can financially save by vaping, so even if they are not sure they like it, if they see/hear that they are saving money they are likely to carry on’* (Ob2)*.* Shops displayed promotional adverts and offered deals such as loyalty cards and multi-buy offers, which vapers appreciated: *‘they’re five pound each juices and they do three for ten pounds, so I always get three’* (F59)*.*

Shop staff acknowledged that vapers often *‘buy online where it’s cheaper’* (Ob5). Vapers described advantages to purchasing online including reduced cost, customer reviews, subscriptions, and a second-hand market. However, unlike online stores, the shops offered instant access to essential consumables: *‘if I’m about to run out [of liquid] then I’ll buy it from a shop, if it’s going to last me another week I’ll buy it off eBay cos it’s a bit cheaper’* (M49). Access to consumables may be crucial in preventing tobacco lapse [33]. The growing number of shops meant more competition: *‘although we have a handful of customers that are really loyal most people will go where the prices are good’* (Ob5). Familiarity was important to novice users establishing vaping, but those with more confidence shopped around: *‘I went to the same shop to begin with, but then as more cropped up around the area, I thought right, let’s go see what these guys have got’* (M53).

#### 3.1.3. Regulation

It was clear that the TRPR brought commercial challenges to the shops, such as ensuring stock was compliant and refitting shop shelves to house smaller bottles. One staff member commented that: *‘they still have hundreds or thousands of pounds of non-TPD compliant products in storage that they are unable to sell’* (Ob3). Staff discussed being uncertain about some aspects of the legislation around devices, especially drippers, with some shops still stocking them and others taking the decision to no longer sell them. Labelling of vaping hardware as containing nicotine seemed nonsensical to vapers familiar with the legislative changes and the shops: *‘it seems strange to give a nicotine warning on products that don’t actually contain nicotine’* (Ob5). Shop staff, who had to explain the legislation to customers, felt it was confusing them and in some instances had resulted in hostility from angry customers upset that non-compliant products were no longer available.

As a reaction to liquid bottle size restrictions some vapers were investigating making their own liquid to avoid the increased cost of buying liquids in smaller amounts. Five of the shops appeared to embrace the home mixing trend, stocking products such as flavour shots which could be added to home mixed liquid, and nicotine shots which could be added to larger sized zero nicotine bottles. These shops were happy to provide ongoing support and advice: *‘because some of [their customers] were skint, some were on the verge of smoking, so [mixing] is good for keeping people excited and keeping down the cost’* (Ob2). Shops felt the smaller bottles encouraged people to experiment with flavours also adding to the excitement of vaping. Overall, it appeared that the shops had adapted to the legislative changes for the most part. 

#### 3.1.4. Health Professional Referral

Traditional smoking cessation support was not perceived as the main role of the shops by either staff or vapers. Yet shops discussed promoting the health benefits of switching to e-cigarettes. For example, one shop displayed a poster outlining why vaping is better for health than tobacco [44]. Some of the shop staff had an awareness and interest in research. For example, the 2015 Public Health England report was mentioned by a couple of staff who had relayed the finding ‘e-cigarettes are around 95% safer than tobacco’ to customers [14] (p. 6).

Although one shop assistant feared that medicalisation would negatively affect consumer freedom, most shops were interested in working closer with health professionals. One shop had received patient referrals because *‘according to the shop assistant, the shop owner took the time to meet with the local GP (family doctor) when he first opened the store and the GP was “fully on-board”* (Ob3). A couple of quitters had also heard of similar informal arrangements between health professionals and shops:

*I said to [shop assistant] “look I’d seen the stop smoking service and that” and he said “have you seen <SSS advisor>?” and I was like “yes I have” and he said “yes she comes in here occasionally and she comes and she talks to us”. So it was almost like that she was taking the time to go out visit the retailers, find out what they were offering.*

(M46)

Nearly all participating vapers wanted the National Health Service to promote e-cigarette use including more information for GPs and even e-cigarettes available on prescription. Interestingly though, around a third of vapers interviewed planned to stop using e-cigarettes. E-cigarette cessation could be in conflict with vape shops’ commercial interests and needs to be explored further, especially given that partnership working between shops and health professionals appears to be increasing.

### 3.2. Interpersonal Environment—Creating a Shared Social Vaping Experience

#### 3.2.1. Friendly Personal Service

A friendly personal service was commented on by vapers and witnessed during the observations. Most of the shop assistants made an effort to be friendly and take a personal interest, but were also sensitive to customers who seemed in a rush or didn’t want to engage in chat. One shop assistant commented that: *‘it is the customer service the managers want and is why the chain is succeeding when others are going under’* (Ob4). This approach was very inclusive of *‘all types of vapers from beginners to enthusiasts’* (Ob3) and seemed to make commercial sense to shop staff who were aware of building a customer base through reviews and retaining customers long-term. This also benefitted vapers through developing trust and relationships with staff who could offer ongoing support to prevent future relapse.

#### 3.2.2. Socialising and Relaxation

Two types of customers were observed during the visits; those who bought consumables and left, and those who stayed to vape and chat seemingly using the vape shop as an opportunity to socialise and relax. Five of the six shops had a similar ‘café feel’ interior with bar stools at the counter, chairs and sofas, a coffee machine, and snacks available to buy. One shop even had *‘a fake fireplace and two leather chairs, plus a chess table’* (Ob5), attempting to encourage customers to stay longer. Much of the conversation observed between staff and customers was ‘vaping talk’ discussing devices, home mixing, and vaping anecdotes. The conversations also strayed onto non-vaping topics including cars, workplaces, health, children, local gossip, and socialising plans. A few vapers discussed enjoying this informal atmosphere: *‘you can go in and sit down and have a coffee, there’s a coffee machine, and have a little vape, quite friendly, quite helpful’.* (F33)

This sense of community could be supportive for quitters, and may be especially appealing to those who enjoyed the social aspect of smoking. However, this café style did not appeal to all vapers interviewed:

*I’ve seen various vaping shops almost like trying to encourage your café atmosphere, none of them have succeeded. You walk in, you buy your liquid, you bugger off, you know. If someone was to set up sort of like a vaping type café, good luck to them, I’m not sure I would use it.*

(M53)

One shop did not have a café feel and instead had *‘a familiar high-street store feel, it is recognisable as a shop that sells consumer goods’* (Ob2). This may potentially be more appealing to vapers, like the person quoted above, who require only access to products and could be less intimidating for someone new to e-cigarettes.

#### 3.2.3. Vaping Identity Reinforcement

Shop staff discussed how there were generally two types of vapers (also identified in previous research [10]); those who used e-cigarettes primarily to replace or cut down smoking, and those who were vaping as both a replacement for smoking and also a hobby, investing greater amounts of time and money into building and modifying devices, making the best-flavoured liquids, or performing cloud blowing tricks. For the latter, spending time in the shop with likeminded people reinforced their vaper identity. For some vapers, the culture presented by the shops was intriguing and exciting, and a couple of vapers described how shop staff had ignited their interest in vaping as a hobby, as well as a replacement for smoking: *‘I had another world was opening up to me and I thought I’d really like to try it and I could see that [shop staff] were really enthusiastic and yes I wanted to join in with it really’* (F34)*.*

For those enjoying vaping as a hobby, vape shops created a feeling of belonging and reinforced a sense of identity. However, other vapers felt that shop staff were not interested in them because they did not identify in the same way and were only interested in people using more advanced setups. Most vapers interviewed saw the vaping culture presented in shops as strange and not relevant to them:

*I opened the door [of the shop] and I just thought oh my god. They’ve got these big square things, the place is on fire! That don’t do nothing for me. I call them serious vapers. It might sound really silly, but I don’t know if they do it for different reasons, that’s how that comes across to me, that to sit there and [vaping noises] and then fill the room up with vape. It’s like going into a smoking room in an airport, which I used to find absolutely vile, and that’s what these shops have become.*

(F60)

Shop observations and interviews with vapers revealed that different shop environments seemed to appeal to different people, but most vapers interviewed had to discover this for themselves through trialing different shops.

#### 3.2.4. Masculine Territories

Regardless of who the shop appealed to, the shops observed did seem to be largely masculine territories. Nearly all the shops had more of a ‘masculine looking’ interior with hard colours and metal finishes. All the participating shop staff, bar one, were male, and by far the majority of customers who stayed to vape and chat were male: *‘at this point the shop feels very much like a traditional pub with men joking and discussing hardware and vaping’* (Ob4)*.* A small minority of female vapers did mention their gender as a reason for not feeling confident in vape shops: *‘I had a feeling [in the shop] of being a woman going into the car mechanics, like they assume you don’t know, so you get fleeced’* (F36b). Three women discussed having their male partner or colleague visit shops on their behalf and, in three shops, male customers were observed buying products and asking advice for their absent female partners. Although this may have simply been for convenience, it is of note that no women were observed doing this for male partners. One vaper felt that the hobbyist aspect of some of the shops was especially off-putting for women:

*I still find [shops] a little bit intimidating because [the shop] I go to they also sell like all the heavily modded tanks and batteries and stuff like that, so they are still a little bit kind of ‘boys clubby’ to me [...]. It’s blokes with their massive batteries and stuff like that, so I just kind of go in and go “right I want that liquid in that strength” and kind of take my leave.*

(F38)

### 3.3. Individual Level Environment—Ensuring a Satisfying and Functioning Vaping Setup

Most quitters did not achieve abstinence with their first device because it did not provide a sufficient nicotine hit to tackle cravings or did not work properly due to user error or malfunction. Therefore, it is helpful if shops are able to offer a supportive environment ensuring that quitters have a satisfying e-cigarette set-up and provide ongoing support for issues ensuring the device continues working properly.

#### 3.3.1. Advice and Navigating Choice

Navigating product choice was an issue for many vapers who described feeling overwhelmed by the product range and, for a few, this had been a barrier to trying e-cigarettes. A couple of vapers had purposefully limited their choice by deciding to buy in supermarkets and pharmacies instead: *‘[vape shop] would be introducing the complexity that I don’t want, I want to keep it simple’* (F62b). Whilst this may make purchasing less confusing, some vapers found limiting product options prompted relapse because the device was less suited to their needs:

*If you are serious about [quitting], I don’t think just buying, like I got the one from the factory shop. It was only like a tenner, it just wasn’t enough. I think you’ve probably got to get a bit of advice, cos it just didn’t work for me.*

(F29)

The shops had organised their devices into ‘beginner’ ‘intermediate’ and ‘advanced’ displays, in an effort to be less confusing. One shop had *‘devices on display but tied by elastic to the counter, they have adopted the mobile phone shop method of display’* (Ob2), allowing the customer to feel the devices. Verbal support appeared most useful in helping vapers navigate this potentially confusing area. Advice to customers looking to quit smoking related to nicotine hit, usability and flavour.

A satisfying physiological experience and reduction in craving was identified by vapers and shops as the fundamental reason for e-cigarette success:

*[Shop assistant] actually took the time to ask me some pertinent questions like “when you smoked your cigarettes, did you breathe direct to lung or did you breathe it into your mouth and then kind of breathe it down?” I quickly found out afterwards these questions are really quite important to getting the right sort of e-cigarette, because if you get the wrong it’s not going to satisfy the need and the chance are that you’re more likely to start smoking again.*

(M46)

Most of the shops discussed ‘rules of thumb’ when recommending nicotine strengths. Generally those smoking up to 10 cigarettes a day were recommended 3–6 mg nicotine, 10–20 cigarettes a day were recommended 6–12 mg, and 20+ cigarettes a day were recommended 18 mg. Vapers liked this approach as it bridged the gap between their familiar smoking habit and their new vaping habit, offering them reassurance that their nicotine needs would be satisfied by switching:

*I just found [shop assistant] to be incredibly helpful. She asked us more about our smoking history than actually I got asked at the smoking cessation clinic. She identified what it was we were smoking at the time, how much we were smoking, and then sort of looked at different strengths of the flavours what would suit us best. She recommended that I started on 18 mg strength nicotine liquid.*

(M44)

The ability of a vaper to operate the device long-term was highlighted by shops as important. Shops discussed finding out how ‘tech savvy’ customers were and would take time to explain how to operate and maintain devices: ‘*we would go through all the information about charging and coils because it can seem quite daunting and if you get one thing wrong then it can mean that it won’t work* (Ob6)*’.* Discussion about battery life was seen as important by vapers and shops:

*[The shop] were helpful in terms of finding something useable and reliable and had a big battery in it so you don’t have to constantly charge it, which is very important actually because I forget to charge it and then I’m in trouble because I don’t have my fall back.*

(M39)

All shops had battery safety leaflets accompanying purchases. One shop assistant commented that: *‘they will rewrap batteries for free because they want vaping to be as safe as possible’ and ‘they always give free cases and bags for batteries’*. This concern about safety seemed to stem from a genuine desire to make vaping safe for customers, but also as an effort to protect vaping’s reputation against negative media reports: *‘the managers are very safety conscious and think that people not taking proper precautions have given vaping a bad name’* (Ob4)*.*

The taste of e-cigarettes was experienced by nearly all vapers as more pleasurable than tobacco and was highlighted by many as a key reason for sustained smoking abstinence. This was reiterated by the shops. For example, one shop assistant said*: ‘that most customers came to enjoy the flavours and got into vaping as being different from smoking, so if relapsed, found they hated the taste of tobacco and would probably go back to the vape’* (Ob5). The shelves displayed many different flavours that people could try and a few shops had menus listing different brands. The staff were also able to make flavours seem exciting providing vivid description: *‘[shop assistant] lets customers try “Kendal mint cake” which he says “reminds me of forest marches across Dartmoor”’* (Ob4). Vapers described how shops enabled them to try different flavours which kept vaping exciting promoting continued use:

*They had a whole sort of display thing and they said “here’s all the different flavours that we sell, try some”. It was a case of just going through and trying and I just didn’t like the flavour of the tobacco ones, I just preferred the fruit flavours. Obviously when you stop smoking you start to get your taste back, so you could actually taste it more and more as time went on, so you were kind of “actually this is quite nice” and then you’d start to experiment with all the different flavours.*

(M46)

The customer service in the shops observed was attentive and helpful. However, the vaper data showed that some had experienced poor service and advice. This mainly related to insufficient product information and shops focused on making a sale:

*They didn’t offer much advice or information. They just had the e-cigarettes and the liquids and said this flavour’s nice, and this flavour’s nice, and there wasn’t really much conversation or information about it. It was very much like they just kind of wanted a sale on it.*

(F25)

#### 3.3.2. Ongoing Support

Ongoing support was highlighted by both shops and vapers as important in ensuring that e-cigarettes continued to function well over the long-term:

*I’m very reliant on going to the shop and going “help something has gone wrong”. They’ll just tut and go “you just do this like”. So I’ve got that reliance, I’ve almost got like a little help on hand.*

(F36a)

There were plenty of instances where the shops gave support to customers who were having difficulties. Several customers asked shop staff to change coils and there were examples of hardware issues being investigated for free. One shop assistant described how he would ‘troubleshoot’ with the customer if they had relapsed and try and find a solution, such as fixing their device or upping their nicotine strength:

*When they relapse find out why... “usually this is stress or because of drinking alcohol, sometimes because their coil started burning or liquid started leaking…. We can help with all those sorts of things”. I ask how he advises about stress or a night out, “that can be a good time to use the device more or maybe go up a level of nicotine”. He also tells me that “most people think their device is broken and they have relapsed, but if they bring it in and show us then we can fix it”.*

(Ob2)

## 4. Discussion

Vape shops offer ‘expert by experience’ product advice to smokers looking to transition to vaping and on-going support to vapers. To date there is little UK research on influential shop environmental factors for supporting quit attempts. This study addressed this by triangulating qualitative data collected through vape shop observations and quitters’ experiences of vape shops. Quitting smoking is a difficult and complex process [7]; high levels of relapse are testament to the addictive potential of tobacco smoking. Given the complexity and challenge, the Royal College of Physicians advises that smokers quitting by using e-cigarettes are likely to need additional behavioural support [13]. This research indicates that vape shops are well placed to offer this type of support.

At an individual level, shops can provide a supportive environment by helping quitters to navigate product choice, thereby ensuring a satisfying hit and a device/e-liquid setup tailored to individual need and based on current smoking behaviour. Shops also provided ongoing support to reduce risk of smoking lapse due to user error, device malfunction and in some cases, because of lifestyle or circumstance changes, (e.g., stress, nights out). Shops supported continued use of e-cigarettes by offering ongoing ‘without sales’ free advice, trouble shooting and support, which in turn promoted a returning customer base. 

At an interpersonal level, shops can offer friendly personable service, creating a relaxing environment, putting novice users at ease and helping them to feel confident about their purchasing choices. For some vapers, shops also provided an opportunity for social interaction, acting as a community group, creating a temporary sense of belonging, reinforcing a vaper identity, motivating people to return to the shop and continue their e-cigarette use. However, we did receive some reports that these environments were perceived as intimidating or off-putting, especially by women and some vapers using e-cigarettes primarily for medicinal use. Nevertheless, there was evidence that the market was evolving to accommodate these groups, for example, via the high-street style shop observed. 

At a structural level, shops provide a competitive environment, responding to legislative changes such as the TRPR. The challenge for shops in adapting to legislative changes was to ensure continued easy access to products, whilst maintaining the financial benefits of transitioning to vaping. Consumers like the non-medicalised environment and the peer support offered in shops by ‘experts by experience’ which may be lost with too much regulation [45]. 

While we observed some rejection of the medicalisation of products and the streamlining of advice, informal co-working between shops and SSS could be particularly helpful for smokers unsure about e-cigarettes or hesitant about using shops. Especially as guidance suggests that combining e-cigarettes with behavioural support offered by SSS has the potential to increase success rates [16]. Health professionals should consider engaging with the local vaping community to avoid referring clients to shops offering poor customer service and inappropriate sales driven advice. However, many quitters do not access SSS support. Furthermore, vape shops provide opportunity to those who do not wish to stop smoking to try vaping and perhaps eventually stop smoking. Therefore, some smoking cessation training for shops (e.g., [46]) could be beneficial [9,47], along with making customers aware of available research resources (e.g., [44,48]). This would ensure that information given about quitting smoking (in addition to product advice) was evidence based. 

### Limitations

The qualitative nature of the study means the findings cannot necessarily be generalised to the wider vaper or vape shop population. However, the data presented is triangulated from two sources. Furthermore, the advice witnessed as being given by shops mirrors advice given by experienced vapers in other research [49]. Further research could investigate if the advice given is quantifiably effective. Although there was diversity in both the observation and interview samples, the most deprived areas and populations (e.g., homeless) were not represented. It is important to acknowledge that poorer communities will be at a disadvantage as they have less access to reputable vape shops and quality products [50].

## 5. Conclusions

Vape shops offer an important ‘expert by experience’ role in supporting smokers to quit. Different shops may appeal to different groups of smokers and vapers, and may encourage those who do not intend to quit smoking eventually to go on to quit. For many, vape shops are an easy to access, non-medicalised source of advice and support. However, some vapers using e-cigarettes mainly for medicinal purposes and some female users found vape shops could be intimidating, and for some communities access to reputable vape shops may be more limited than in the areas where this research was carried out. Future research could consider evaluating joint working between Stop Smoking Services and vape shops to help smokers achieve and maintain smoking cessation.
